# Draft genome sequence of *Salmonella enterica* subsp. *enterica* serovar Typhimurium SBI_US10_MRI_BD isolated from broiler chicken in Bangladesh

**DOI:** 10.1128/mra.01203-25

**Published:** 2026-02-27

**Authors:** Md Raihan Islam, Tamanna Hossen, Md. Safayat Sahib, Anjala Barua Anne, Esrat Jahan, Tahrima Anzum, Raiyan Ul Hakim, Tahrima Saiha Huq, Kazi Rahman, Md. Hafizur Rahman, Kazi Fahmida Rahman, Kazi Samia Pial, Md. Fakruddin, Spencer Mark Mondol, Md Murshed Hasan Sarkar, S. M. Bakhtiar UL Islam

**Affiliations:** 1 Department of Biochemistry and Microbiology, School of Health & Life Sciences, North South University 54495, Dhaka, Bangladesh; 2 Department of Microbiology, University of Dhaka 95324, Dhaka, Bangladesh; University of Maryland School of Medicine, Baltimore, Maryland, USA

**Keywords:** Bangladesh, broiler chicken, BV-BRC, PGAP, *Salmonella enterica*

## Abstract

*Salmonella enterica* is a rod-shaped, gram-negative bacterium classified under the *Enterobacteriaceae* family. Herein, we report the 4,918,555 bp genome (50.08% G+C) with 272.5× genome coverage of *S. enterica* SBI_US10_MRI_BD, containing a total of 4,754 CDSs, 55 tRNA, 2 rRNA, and 14 ncRNA genes.

## ANNOUNCEMENT

*Salmonella* spp. are gram-negative bacilli (*Enterobacteriaceae*) widely found in the intestine of animals and humans (1, 2). As a significant zoonotic pathogen, transmission to humans frequently occurs via the fecal-oral route through contaminated animal-derived foods, particularly poultry and meat products (3). To understand the growing frequency of multidrug resistance (MDR), genomic surveillance of broiler-derived bacterial strains has become essential.

*Salmonella enterica* SBI_US10_MRI_BD was isolated in 2024 from cloacal swabs of processed broiler chickens from Uttara Sector-12 (latitude: 23.8714°N, 90.3807°E), Uttara, Bangladesh. Briefly, samples (*N* = 12) were collected aseptically using sterile cotton swabs and placed into 15 mL sterile tube containing 3 mL buffered peptone water (BPW) as a transport and pre-enrichment medium. Samples were then transported to the Microbiology laboratory at North South University and incubated in a shaking incubator (150 rpm) at 37°C for 18 h. After 10-fold serial dilution, single colonies were isolated on selective Salmonella-Shigella agar. Pure cultures were streaked onto nutrient agar, and 12 isolates were preserved in glycerol at −20°C.

Presumptive identification was performed using Gram staining and standard biochemical tests, including TSI, Simmons citrate, MR-VP, catalase, oxidase, MIU, and nitrate reduction assays. Antibiotic sensitivity testing was conducted using the Kirby-Bauer disk diffusion method. Among the isolates, we performed whole-genome sequencing (WGS) on the multidrug-resistant *S. enterica* strain SBI_US10_MRI_BD to determine its serovar, antimicrobial resistance, virulence profiles, and genomic features relevant to public health surveillance.

For WGS, the *S. enterica* SBI_US10_MRI_BD was cultured overnight on nutrient agar at 37°C. The isolate was submitted for WGS to the Genome Sequencing Laboratory, BCSIR, Dhaka, Bangladesh. Genomic DNA extraction and library preparation were performed as per the manufacturer’s instructions using Qiagen DNeasy Blood & Tissue Kit (Cat. 69504, Hilden, Germany) and Nextera DNA Flex Library Preparation Kit (Cat. 20018705, Illumina, San Diego, USA), respectively. Sequencing was carried out on the Illumina NextSeq 550 platform, generating Illumina paired-end raw 150 bp reads, as previously described (4, 5).

Genome quality assessment was performed using the workflows provided by the Bacterial and Viral Bioinformatics Resource Center (BV-BRC) with default parameters. For quality control, Trim Galore version 0.6.5dev (6, 7) was used to trim paired-end reads, and *de novo* assembly was conducted using Unicycler (v0.4.8) (8), implemented within the BV-BRC pipeline (v3.46.3) (9) using the default parameters except where otherwise noted. Assembly quality metrics, including coarse consistency (99.2%), fine consistency (98.7%), contamination (1.2%), and CheckM completeness (100%), indicated a high-quality genome assembly. The total raw reads of the original sample contained 4,463,482 reads (319.15 million bases) with an average read length of 35.8. The final genome size was 4,918,555 bp, with an average G+C content of 50.08% and an average sequencing coverage of 272.5×. The draft genome assembly comprises 71 contigs (>300 bp), with an *N*_50_ of 157,886 bp and an *L*_50_ of 12.

Genome annotation for *S. enterica* SBI_US10_MRI_BD was performed using the NCBI Prokaryotic Genome Annotation Pipeline (PGAP) version 6.10 with default parameters (10). The annotated genome contained a total of 4,754 CDSs, 55 tRNA genes, 2 rRNA genes, and 14 ncRNA genes.

## Data Availability

The data described in this report are publicly accessible through NCBI BioProject PRJNA1313641 and BioSample SAMN50904865. The draft genome sequences have been deposited in GenBank under SRA accession number SRR35210187. This Whole Genome Shotgun project has been deposited at DDBJ/ENA/GenBank under accession JBQWDY000000000. The version described in this paper is version JBQWDY000000000.1.

## References

[1] Brenner FW, Villar RG, Angulo FJ, Tauxe R, Swaminathan B. 2000. Salmonella nomenclature. J Clin Microbiol 38:2465–2467. doi:10.1128/JCM.38.7.2465-2467.2000 10878026 PMC86943

[2] Eng S-K, Pusparajah P, Ab Mutalib N-S, Ser H-L, Chan K-G, Lee L-H. 2015. Salmonella: a review on pathogenesis, epidemiology and antibiotic resistance. Front Life Sci 8:284–293. doi:10.1080/21553769.2015.1051243

[3] Majowicz SE, Musto J, Scallan E, Angulo FJ, Kirk M, O’Brien SJ, Jones TF, Fazil A, Hoekstra RM. 2010. The global burden of nontyphoidal Salmonella gastroenteritis. Clin Infect Dis 50:882–889. doi:10.1086/650733 20158401

[4] Raida AI, Sahib MS, Hakim RU, Raihan J, Tayeb MA, Fakruddin M, Mondol SM, Islam SMBU. 2025. Draft genome sequence of Bacillus cereus SBI_SS06_AR_BD: a bacterium producing both alpha-amylase and lipase. Microbiol Resour Announc 14:e0026725. doi:10.1128/mra.00267-25 41059688 PMC12613945

[5] Anne AB, Hossen T, Sahib MdS, Hakim RU, Islam MdR, Supty SM, Saadat TMdF, Islam KT, Rubaiyat RN, Fakruddin Md, Mondol SM, Islam SMBU. 2025. Draft genome sequence of Salmonella enterica subsp. enterica serovar Typhimurium SBI_BS16_ABA_BD isolated from broiler chicken in Dhaka, Bangladesh. 2025. Microbiol Resour Announc 15:e01175-25. doi:10.1128/mra.01175-25 41288074 PMC12797928

[6] Krueger F. 2012. Trim galore: a wrapper tool around cutadapt and fastqc to consistently apply quality and adapter trimming to FastQ Fles, with some extra functionality for mspi-digested rrbs-type. Available from: http://wwwbioinformaticsbabrahamacuk/projects/trim_galore

[7] Bolger AM, Lohse M, Usadel B. 2014. Trimmomatic: a flexible trimmer for Illumina sequence data. Bioinformatics 30:2114–2120. doi:10.1093/bioinformatics/btu170 24695404 PMC4103590

[8] Wick RR, Judd LM, Gorrie CL, Holt KE. 2017. Unicycler: resolving bacterial genome assemblies from short and long sequencing reads. PLoS Comput Biol 13:e1005595. doi:10.1371/journal.pcbi.1005595 28594827 PMC5481147

[9] Olson RD, Assaf R, Brettin T, Conrad N, Cucinell C, Davis JJ, Dempsey DM. 2023. Introducing the bacterial and viral bioinformatics resource center (BV-BRC): a resource combining PATRIC, IRD and ViPR. Nucleic Acids Res 51:D678–D689. doi:10.1093/nar/gkac1003 36350631 PMC9825582

[10] Tatusova T, DiCuccio M, Badretdin A, Chetvernin V, Nawrocki EP, Zaslavsky L, Lomsadze A, Pruitt KD, Borodovsky M, Ostell J. 2016. NCBI prokaryotic genome annotation pipeline. Nucleic Acids Res 44:6614–6624. doi:10.1093/nar/gkw569 27342282 PMC5001611

